# Workplace Bullying and Post-Traumatic Stress Disorder Symptomology: The Influence of Role Conflict and the Moderating Effects of Neuroticism and Managerial Competencies

**DOI:** 10.3390/ijerph191710646

**Published:** 2022-08-26

**Authors:** Miren Chenevert, Michela Vignoli, Paul M. Conway, Cristian Balducci

**Affiliations:** 1 Department of Psychology, Alma Mater Studiorum–Università di Bologna, 40127 Bologna, Italy; 2 Department of Psychology and Cognitive Science, University of Trento, 38068 Rovereto, Italy; 3 Department of Psychology, University of Copenaghen, 1353 Copenaghen, Denmark

**Keywords:** role conflict, workplace bullying, PTSD symptomology, neuroticism, managerial competencies

## Abstract

Research has explored numerous consequences of workplace bullying, including a recent link to the exhibition of post-traumatic stress disorder (PTSD) symptomology. Role conflict as a workplace stressor may contribute to instances of bullying from a passive perspective, which may lead to PTSD symptomology in victims. What remains less explored is if role conflict has a direct relationship to PTSD symptomology and how personality traits such as neuroticism and workplace factors such as managerial competencies may moderate the stress brought on by role conflict. Hence the present study seeks to examine this gap in the literature. This study utilizes a between-subjects, cross-sectional design with 159 participants, 39.6% male and 60.4% female. Most participants (60%) were Italian workers of a large social cooperative organization. Confirmatory factor analysis indicated that the measurement model was valid and had an adequate model fit. Results from two separate moderated mediation analyses found a positive, full mediation between the independent variable of role conflict, the mediator of exposure to bullying, and the dependent variable of PTSD symptomology. Furthermore, in this study, neuroticism strengthened the indirect effect while managerial competencies weakened it. The results highlight the importance of training competent managers and providing resources for more vulnerable employees to moderate employee work stress and its negative outcomes.

## 1. Introduction

Bullying and its effects have been studied extensively by sociologists and psychologists for decades. Typically, bullying is most prevalently studied and discussed within the context of schools, with children as the subjects. These studies have found that subjects of bullying report a variety of negative physical, social, and psychological effects [1]. While school bullying is universally recognized, bullying in the context of the adult workplace is rarely considered. Nonetheless, beginning in the 1990s, models and frameworks have been developed to conceptualize the process of workplace bullying using the limited research available [2]. Workplace bullying refers to a situation where one or several individuals, persistently and over time, perceive themselves to be the victim of negative actions from one or several persons [2]. In 2010, the EWCS (European Working Conditions Survey) found that 14% of all European workers had reported experiencing at least one form of ASB (adverse social behavior) in the workplace. In addition, Italy reported values above the European average for instances of bullying and harassment. Information from the national contributions showed that verbal and psychological aggression—ranging from threats, intimidation, verbal abuse, bullying, harassment, mobbing, and psychological violence—constitute the most reported forms of ASB in the EU [3]. A survey conducted later in 2015 by EU-OSHA showed comparable results, with 4% of all European workers reporting incidences of bullying specifically, with women reporting bullying more frequently than men overall. Instances of bullying in Italy were particularly high in the healthcare sector, with nurses reporting higher instances of workplace bullying in the medical field, especially by superiors. Scandinavian countries such as Finland, Sweden, and Norway reported the highest occurrences of workplace bullying, with numbers more than triple that of the European average (13%) [4].

The European Union is not alone in reporting the prevalence of workplace bullying. In 2019, Forbes published an article detailing the prevalence of workplace bullying in the United States that reported that four out of ten American professionals have been bullied at work [5]. Furthermore, this statistic has increased by 19% over the last eleven years [5]. In 2008, a report commissioned by the Dignity at Work Partnership reported that workplace bullying in the UK costs society approximately GBP 682.5 million annually as society must bear certain monetary consequences of workplace bullying, such as medical and psychological treatment expenses, early retirement, and employees′ benefits [6]. Apart from the expenses, workplace bullying costs organizations 33.5 million workdays due to bullying-related absenteeism and 100 million days of productivity due to bullying-related turnover [6]. Moreover, bullying-related litigations, brand image damages, early retirement, absenteeism, productivity loss, and turnover training monetarily cost UK organizations approximately GBP 13.75 billion a year [6].

In addition to direct organizational losses such as turnover and absenteeism, workplace bullying can cause companies to lose cognitive capital. In the age of entrepreneurial startups and globalized businesses, creativity and innovation are becoming the most desired employee skill sets [7]. Furthermore, the COVID-19 pandemic has demonstrated that companies with creativity and adaptability have an advantage over their competitors, exemplifying that creativity may make the difference between business success and failure in uncertain times [7]. Studies have found that victims of bullying, particularly those who are victims of supervisor bullying, demonstrate less creativity and innovation in the workplace. Psychological safety, a key component of fostering creativity, is lost when bullying is a part of the work environment, especially when managers are the perpetrators. Workplace bullying has been related to risk aversion in victims, signifying that bullying victims will avoid uncertainty and risk-taking for fear of the consequences, thus hindering their creative capacity [8].

Organizational awareness of their role in creating environments that cultivate bullying is imperative to finding effective interventions to combat workplace bullying. In fact, effective measures against workplace bullying are a research gray area. A rapid evidence assessment of studies measuring the effectiveness of interventions for workplace bullying found little to no quality evidence that the current interventions for workplace bullying are effective [9]. The current interventions most used to combat workplace bullying are all tertiary techniques, namely, conflict moderation or mediation and coaching, which address the interpersonal component of bullying rather than organizational components [10]. In parallel, Psychiatrist Vivien Kemp [11], who works with both victims and perpetrators of bullying, expressed that there is still no strong evidence for the most effective remediating interventions for workplace bullying. The most effective interventions tackle the source of the problem rather than the symptoms [12]. Thus, to design interventions that effectively address the root of workplace bullying, it first must be understood how workplace bullying starts and which workplace variables may contribute to its escalation or control. The aim of this study is to identify an organizational antecedent of workplace bullying as well as moderators to this relationship while further investigating the consequences of workplace bullying.

Workplace bullying has a multitude of definitions in the literature and can also be referred to as mobbing or workplace harassment. The concept can be summarized as the repeated mistreatment of an employee by the same individual(s) over time, regardless of the position, intent, overtness, or physicality of the act [13]. Consequently, workplace bullying may be viewed as a continuum of recurring actions that range from more subtle behaviors such as microaggressions to more pronounced acts such as sexual harassment or systemic discrimination [13]. Research has categorized bullying behaviors using three factors that respond to the relation, nature, and tangibility of the act: work/person-related, passive/active, and physical/psychological [14]. The first factor refers to mistreatment related to the victim′s work or to their person [14]. The assignment of meaningless tasks or unmanageable deadlines are forms of work-related bullying, while teasing, spreading rumors, and insulting an individual are types of person-related bullying [14]. Additionally, the nature of bullying, whether passive or active, is another category [14]. Passive bullying refers to indirect behaviors such as excluding someone from group events, while active bullying refers to direct behaviors such as face-to-face verbal aggression [14]. Lastly, bullying can be categorized as physical or psychological, referring to the tangibility of the act. Physical bullying involves tangible actions such as repeatedly touching a coworker inappropriately without consent, while psychological bullying includes intangible actions such as assigning meaningless tasks, teasing, or spreading rumors. Furthermore, bullying differs from other types of office discord due to the continuity of the mistreatment. To be defined as bullying, the mistreatment must take place repeatedly. Single, isolated events of offenses, while harmful, are not incidences of bullying [13]. One verbal argument with a colleague, for example, is simply interpersonal conflict in the workplace. If the same employee were to continuously instigate an argument with the same individual over a period of time, the behavior would be considered bullying.

Experiencing bullying has been linked to several psychological, physiological, and behavioral consequences [15,16,17]. More specifically, workplace bullying has been positively associated with physiological problems such as musculoskeletal health complaints and, most predominantly, negatively associated with psychological health and wellbeing [15,16,17]. In fact, bullied employees have a significant risk for long-term sickness absences, which is detrimental to both the health of employees and the function of organizations [18]. Victims of workplace bullying have reported pervasive emotional reactions such as fear, anxiety, helplessness, depression, irritability, social isolation, and shock [15,16,17]. These emotional responses fit into the three categories of PTSD symptoms defined by the DSM IV-TR (re-experiencing, avoidance, and persistent perception of heightened current threat) [19]. Such connections have led researchers to conclude that victims of bullying at work suffer from symptoms associated with post-traumatic stress disorder (PTSD), not necessarily the disorder itself [15].

PTSD symptoms negatively affect individuals, leading to a multitude of complications such as avoidance, rumination, memory, concentration and sleep problems, dissociation from previously enjoyed activities, self-loathing, and hypersensitivity [19]. While there have been advancements in understanding the relationship between workplace bullying and PTSD symptomology, there has been little investigation of the relationship between possible antecedents of workplace bullying, such as role conflict, and their direct relationship to workplace bullying outcomes, such as PTSD symptomology.

Like bullying, role conflict and other role stressors have direct links to the experience of workplace stress [20]. Workplace stress describes the change in one′s physical and/or mental state in response to workplace situations that pose an appraised challenge or threat, such as being asked to fulfill roles that contradict one another [20]. Role conflict specifically refers to incompatible job demands placed on an employee [20]. Indeed, the workplace situations that contribute to workplace stress include role stressors such as role conflict as well as work overload, lack of autonomy, and a negative organizational climate [20]. If these stressors are continuous, employees are at significant risk of developing physiological and psychological disorders, which, in turn, can lead to organizational consequences such as increased absenteeism, decreased organizational function, and decreased work productivity [20].

Personality factors, while examined as antecedents of bullying, have yet to be investigated as a moderator of the relationship between work stressors such as role conflict and workplace bullying. Research has found that certain personality traits are linked to different coping styles [21]. Coping styles influence how well one manages stress and thus can determine the severity or appearance of stress-induced consequences in an individual, such as workplace conflict and decreased performance [21]. Previous studies have linked neuroticism with maladaptive coping strategies [21]. Poorly managed stress can alter an employee′s mood, making them curt and cynical to their colleagues. More irritable employees are easier bullying targets as bullying behavior against them can be more easily justified [13]. Additionally, the literature has found that managers play a critical role in how well their employees manage workplace stress [22]. Certain competencies possessed by managers have been linked to the stress management of their employees [22]. As role conflict acts as a workplace stressor, this study seeks to further investigate how neuroticism and managerial competencies might affect an employee′s ability to cope with stress brought on by role conflict and thus affect their exposure to bullying.

**Figure 1:**
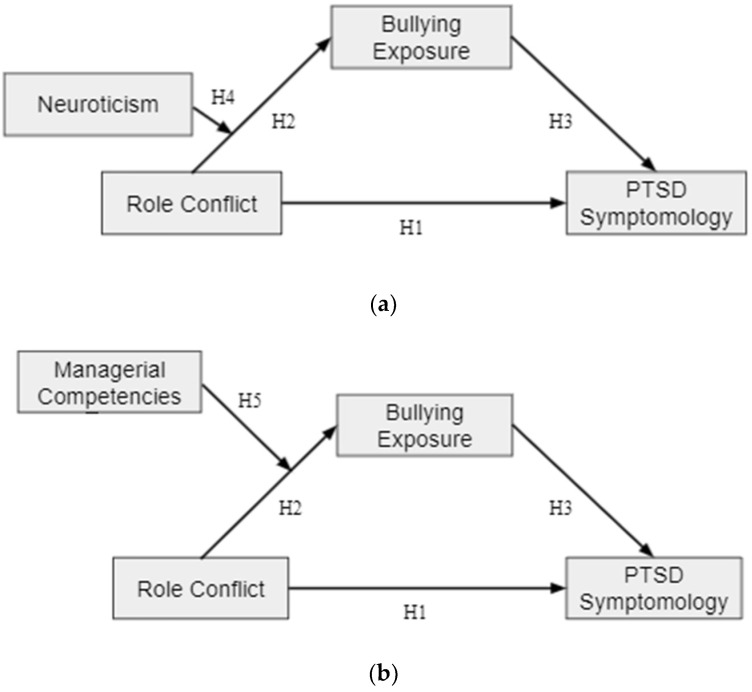
Moderated mediation models. (**a**) Hypothesized moderated mediation model. Neuroticism strengthens the Indirect Effect of Role Conflict and Exposure to Bullying on PTSD Symptomology. (**b**) Hypothesized moderated mediation model. Managerial competencies weaken the Indirect Effect of Role Conflict and Exposure to Bullying on PTSD Symptomology.

This study seeks to provide evidence for effective primary interventions for the prevention of workplace bullying through the exploration of possible at-risk and adaptive groups as well as to underline the importance of the negative impact of workplace bullying on employees through the investigation of PTSD symptomology as a bullying outcome. Thus, the models of this study (pictured in Figure 1) are moderated mediation models, predicting that the relationship between role conflict and PTSD symptomology will be mediated by exposure to bullying and that this indirect effect will be moderated separately by managerial competencies and neuroticism.

## 2. Theoretical Background

**Hypothesis** **1.** *Role Conflict Will Positively Relate to PTSD Symptomology*.

There is a significant overlap between work stress symptoms and PTSD symptomatology. Psychosocial and behavioral consequences such as insomnia, heightened emotions, irritability, fatigue, and emotional desensitization are symptoms of both PTSD and repercussions of work stress brought on by role stressors such as role conflict [19,23]. Theoretical literature tells us that psychological wellbeing rests on the assumption that it is the meanings people assign to role experiences that influence psychological wellbeing rather than factors that influence role strains [24]. Therefore, employees who report greater feelings of role stressors, such as role conflict, should report significantly lower levels of psychological wellbeing [24]. However, a direct link has yet to be found between role stressors and PTSD symptomology. This study will investigate if role conflict alone is enough to cause serious psychosocial symptoms such as PTSD symptomatology or if workplace bullying is a necessary component in this relationship.

**Hypothesis** **2.** *Role Conflict Will Be Positively Related to Bullying Exposure*.

Personal factors involving both the bully and the victim are inherent in the bullying process. However, these behaviors do not occur in a vacuum, and contextual aspects are important for aiding or obstructing the process. Indeed, research has found that organizational culture factors might foster an organizational climate that promotes bullying in the workplace [13]. Einarsen and colleagues [13] found that certain work cultures ignore or even reward bullying and intimidate victims into silence. A work culture that fosters workplace bullying may have no specific protocols for reporting mistreatments, ignore the employee wellbeing component of HR, or have managers that partake in bullying themselves. Ignored or rewarded bullying behaviors occur mainly in bullying incidents related to gender discrimination and sexual harassment as well as between supervisors and subordinates [13]. More concretely, Einarsen and colleagues [13] suggest that organizational factors such as culture and climate are key to determining whether a work environment fosters or hinders bullying.

Other researchers have also examined the role of organizational factors as contributors to workplace bullying, including work-related stressors such as role conflict and job ambiguity. According to the work environment hypothesis, work environment factors are related to bullying, independent of individual factors such as personality [25,26]. These studies found that in work environments where job ambiguity, role conflict, and an imbalance of job demands and resources were present, there was more discord in the office, often escalating to instances of bullying [25]. A Belgian study in 2009 found that role stressors such as role conflict were positively related to exposure to bullying behaviors [27,28]. In addition, role conflict, which describes the situation in which an employee is given incompatible demands relating to their job or position, reduces employee performance, increasing the employees’ vulnerability and their likelihood of becoming a target of bullying [13]. While some research has found that victims of workplace bullying have also reported poor psychosocial work environments, the exclusive effects of role conflict on workplace bullying require further investigation.

**Hypothesis** **3.** *Bullying Exposure Will Positively Relate to PTSD Symptomology*.

Like children, adults are not immune to the effects of bullying. The same negative psychosocial and physical effects that young bullying victims encounter are also experienced by adults. The literature has categorized workplace bullying as a type of work-related stressor. However, research has concluded that the effects of workplace bullying are far more devastating than the effects of all other work-related stressors combined [13]. Therefore, the effects of workplace bullying more closely resemble those of traumatic life events such as domestic and sexual abuse [28,29]. More recently, the individual negative consequences of workplace bullying have been categorized as PTSD symptomology [15]. A PTSD diagnosis refers to a sequence of stress symptoms following a traumatic event or events [19]. As discussed previously, these PTSD symptoms vary by individual but can be grouped into three general areas: re-experiencing, avoidance, and hyperarousal [19]. Re-experiencing includes intrusive memories such as flashbacks to the traumatic event, nightmares about the event or a similar dream event, and emotional stress when confronting a reminder of the event. Symptoms of avoidance include refraining from participating in activities, frequenting places, or speaking with people associated with the event and refusing to speak and/or think about the event. Lastly, hyperarousal includes intense emotional reactions such as being easily startled, always being on guard for danger, self-destructive behavior, alertness that causes trouble with sleeping, difficulty concentrating, irritability, angry outbursts or aggressive behavior, and/or overwhelming guilt or shame [30]. While bullying victims may not fit the full PTSD diagnosis, they may exhibit several symptoms of the disorder mentioned above.

Between-subjects studies have found that compared to controls, those who have been exposed to workplace bullying experience PTSD symptomology [31,32]. While PTSD symptoms vary by individual from moderate to severe, victims of bullying experience negative effects both inside and outside the workplace. A study conducted by Mikkelsen and Einarsen [33] on 118 victims of workplace bullying found, for example, that the majority of the bullied participants experienced impairment in functioning due to hyperarousal symptoms or avoidance behaviors, which affected their work, relationships, sex lives, and even participation in leisure activities such as hobbies. In order to continue to emphasize the importance of investigating workplace bullying, this study seeks to contribute to the evidence that supports PTSD symptomology as a direct consequence of workplace bullying.

**Hypothesis** **4.** *Neuroticism Will Strengthen the Indirect Effect of Role Conflict and Exposure to Bullying on PTSD Symptomology*.

Personality factors, while examined as antecedents of bullying, have yet to be systematically investigated as a moderator of the relationship between work stressors such as role conflict and workplace bullying [34]. Research has found that certain personality traits are linked to different coping styles [35]. Coping styles influence how well a person manages stress and can thus determine the severity or appearance of stress-induced consequences in an individual, such as workplace conflict and decreased performance [35]. The transactional model of stress and coping, which concerns the individual and the environment interaction that forms the appraisal of a stressor and how well it can be managed, may reveal that personality traits act not only as antecedents of bullying but also contribute to how one appraises stress, such as with role conflict [23]. Can personality function as a moderator of the relationship between role conflict and exposure to bullying and thus the exhibition of PTSD symptomology?

A range of studies have found that personality traits can predict the stress level induced by an event. Researchers have found that individuals with maladaptive personality traits, mainly neuroticism, are more susceptible to experiencing negative emotion and frustration from a potential stressor than those with high extraversion and conscientiousness. These personality differences in event appraisal influence the coping style chosen by individuals [36]. Coping is a term used to describe the process in which individuals regulate negative emotions, feelings, and actions elicited by a stressful event and its internal and/or external demands [36]. Previous studies have linked neuroticism with maladaptive coping strategies such as emotional and avoidance coping that refer to the use of disengagement or substances to diminish the negative emotions and the minimization of the stressor through ignorance, denial, and undermining, respectively [37]. Poorly managed stress can alter an employee′s mood, making them curt and cynical to their colleagues. More irritable employees are easier bullying targets as bullying behavior against them can be more easily justified [13]. Further investigation is needed to determine how neuroticism might affect an employee′s ability to cope with stress brought on by role conflict. Thus, the present study will look to fill this research gap by examining neuroticism as a moderator of the indirect relationship between role conflict and exposure to bullying and their effects on PTSD symptomology exhibition.

**Hypothesis** **5.** *Managerial Competencies Will Weaken the Indirect Effect of Role Conflict and Exposure to Bullying on PTSD Symptomology*.

Certain competencies possessed by managers aid in the stress management of their subordinates [19]. One of the ways in which managers can affect an employee′s stress level is through the crossover contagion process [34]. This process describes the way in which supervisors can transmit their level of stress to employees simply by interacting with them through emotional contagion [34]. Other supervisory behaviors such as support and integrity have been found to positively influence aspects of employee wellbeing, such as mental health and job attitudes [34]. Concurrently, the way in which managers organize and manage their employees influences the stress levels and effective coping styles of their subordinates. Managerial competencies that have been found to have a positive link to the stress management of employees can be placed into three categories: altering working conditions, altering environment transactions, and managing individuals within a team [22,38]. The first competency describes the way in which managers can help employees cope with workplace stress by removing obstacles such as work overload, isolation, lack of autonomy, and isolation in order to create the optimal work environment for the employee. The second competency involves the way in which a manager helps an employee cope with stress by improving their interaction with their work environment. This includes actions such as providing employees with services such as employee assistance programs, links to stress management resources, training for behavioral skills, and meditation and relaxation techniques to relieve the physical and psychological effects of stress. The last competency involves how a manager adequately oversees inter-team conflicts in the work setting. This includes skills such as strategic development in tension reduction and proper allocation of individual tasks within a team [22,38]. When a manager possesses these competencies, the literature has shown that their employees cope better with stress [22,38]. As role conflict is a workplace stressor, this study seeks to identify if individuals with managers who possess high-stress management competencies experience less bullying as they can cope with the stress of role conflict better and are thus not subject to stress symptoms that render them vulnerable to bullying, such as decreased performance and irritability.

## 3. Materials and Methods

### 3.1. Study Design and Procedure

To test the hypotheses, the study utilizes a between-subjects, cross-sectional design. The majority of the participants (60%) were Italian workers for a large social cooperative organization. The remaining workers were recruited from a variety of organizations via snowball sampling by directly contacting workers in the network of the researchers. In summary, convenience sampling was the sampling strategy of this study. Participants responded to a questionnaire, establishing a general socio-demographic profile that included variables such as age, gender, job title, and personality traits, as well as measures for accessing the competency of their managers, their exposure to workplace bullying, their experience of role conflict, and their exhibition of PTSD symptomology. The results of this questionnaire were used to conduct moderated mediation analyses to test the hypotheses.

### 3.2. Participants

The sample consisted of 159 participants. Of those 159 participants, all completed the questionnaire. In the sample, 63 (39.6%) of the participants were male and 96 (60.4%) were female. The average age of participants was 36.7 years old, with the youngest participant being 22 and the oldest 63; 59.1% of the participants reported that their job included the coordination of other employees; in other words, more than half of the participants had managerial duties.

### 3.3. Measures

#### 3.3.1. Role Conflict

Role conflict was measured using five items (e.g., “I receive incompatible requests from two or more people”) from the role conflict scale developed by Rizzo, House, and Lirtzman [39]. Responses were chosen from a 5-point Likert scale ranging from 1 (“Entirely true”) to 5 (“Entirely false”), with items being reverse-coded before the scale total was computed. The five-item adaptation of the original scale has previously been used in the Italian context by Balducci and colleagues [40], with a reliability measure of 0.74. The reliability measure of the scale for the present study is 0.76.

#### 3.3.2. Exposure to Workplace Bullying

To measure a participant′s exposure to workplace bullying, the Italian-validated Short Negative Acts Questionnaire (S-NAQ) was used [41]. The S-NAQ was developed by Notelaers and colleagues [14] by adapting both the original NAQ and the later improved version, the Negative Acts Questionnaire Revised (NAQ-R) [27]. The S-NAQ is a nine-item scale with three items measuring each dimension of workplace bullying (i.e., person-related, work-related, and social isolation); it can also be used unidimensionally [14]. Participants report their experience of each item in the last six months (e.g., “Someone withholding information which affects your performance”) using a five-point Likert scale ranging from 1 (Never) to 5 (Daily) [14]. The present study utilizes the Italian-validated version of the tool, where the questions, as well as the Likert scale, have been translated into Italian [41]. The reliability measure of the Italian-validated scale was 0.84 [41]. The overall reliability measure for the S-NAQ in the present study is 0.83.

#### 3.3.3. PTSD Symptomology

PTSD symptomology was measured using an abbreviated version of the PTSD Checklist for Civilians (PCL-C), developed and validated by Lang and Stein [42]. The abbreviated instrument reduced the original 17-item scale into 2-, 3-, 4-, and 6-item scales measuring PTSD symptomatology and severity [42]. The abbreviated versions include three subscales (i.e., re-experiencing, avoidance, and hyperarousal) that investigate the three types of symptoms of PTSD, as defined by the DSM IV-TR [42]. Responses to items (e.g., “I avoid activities or situations because they remind me of a stressful experience from the past”) answered not only the occurrence of the symptom but also how much the symptom disrupted the individual′s life, varying from 1 (Not at all) to 5 (Extremely). The overall alpha for the four-item scale (validated as a unidimensional scale), the scale employed in the present study, was 0.82 [42]. The four-item scale consists of two items measuring re-experiencing, one item measuring avoidance, and one item measuring hyperarousal [42]. The reliability measure of the scale in the present study is 0.81.

#### 3.3.4. Managerial Competencies

Managerial competencies were measured using the nine items (e.g., “My supervisor makes it explicit that he will take ultimate responsibility if things go wrong”) of the Stress Management Competency Indicator Tool developed by Toderi and Sarchielli [43]. Responses were recorded using a 5-point Likert scale, from 1 (completely disagree) to 5 (completely agree). The scale was developed to measure supervisors′ behaviors that have been associated with the reduction of employees′ work stress, which can be divided into three categories: altering working conditions, altering environment transactions, and managing individuals within a team. Each of these subdimensions contain three items. The original scale had a unidimensional reliability measure of 0.85 [43]. The reliability measure of the present study is 0.89.

#### 3.3.5. Neuroticism

Goldberg [44] categorized neuroticism as emotional instability and reactivity. In this study, neuroticism was measured using four items derived from the Italian version of the Big Five scale [45] used in several studies in the Italian context [46]. Balducci and colleagues [46] selected four items from the Flebus [45] personality scale for neuroticism with the highest factor loadings yielded in previous research. The shortened version of the scale consists of four questions (e.g., “I get stressed out easily”) with a 5-point Likert scale ranging from 1 (“It doesn′t describe me at all”) to 5 (“It describes me completely”). In the most recent Italian study applying the four-item scale, the reliability measure was 0.81 [46]. The current study has a reliability measure of 0.83.

### 3.4. Data Analysis

Assumption tests for the moderated mediation models, the reliability analysis, the correlations, as well as the descriptive statistics of the sample were tested using the software IBM SPSS Statistics 22.0. A composite score for each variable (i.e., role conflict, exposure to workplace bullying, PTSD symptomology, neuroticism, and managerial competencies) was computed by adding the respective items of each scale and computing an average score as each scale has been shown to be unidimensionally valid in previous studies. The validity of each scale was confirmed using confirmatory factor analysis conducted using version 23.0 of the Amos statistical software. Factor loadings for the total measurement model are presented in Appendix A. To evaluate the hypotheses, two separate moderated mediation analyses were conducted to test the relationship between role conflict and PTSD symptomology through exposure to bullying, considering neuroticism and managerial competencies as possible separate moderators of the ‘a’ path. The statistical program used to conduct the analysis of the hypotheses was version 3.5 of the PROCESS tool for SPSS, using model seven. Lastly, the accepted established significance level for this study was *p* < 0.05 for all the analyses conducted.

## 4. Results

First, means, standard deviations, and correlations between variables were calculated, as seen in Table 1. Managerial competencies and PTSD symptomology were the only pair of variables that were uncorrelated.

### 4.1. Moderated Mediation with the Moderator of Neuroticism

**Figure 2:**
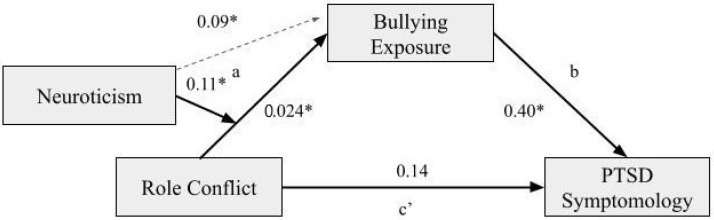
Coefficients of the moderated mediation model with neuroticism as a moderator. *Note.* * indicates *p* < 0.05. c’ (c-prime) refers to the direct effect.

**Figure 3:**
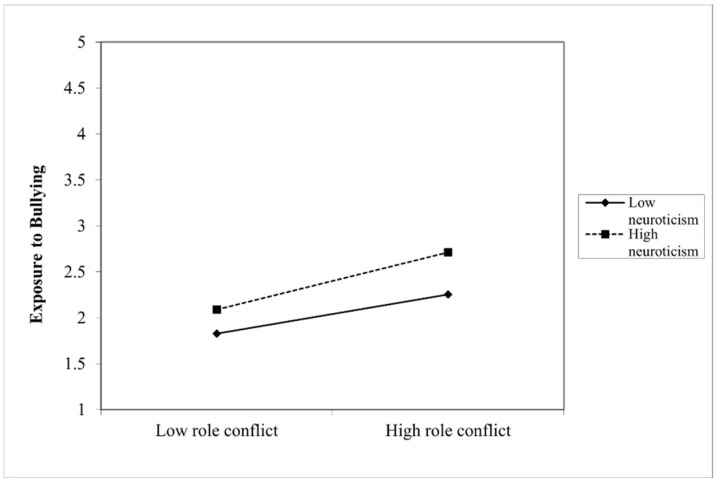
The moderating effects of neuroticism on the indirect effect of role conflict and exposure to bullying.

The analysis found a statistically significant moderated mediation, with the moderator of neuroticism reporting an index of 0.05 and bootstrapping confidence intervals between 0.004 and 0.10. The direct path between role conflict and PTSD symptomatology was not significant, thus showing a full mediation where role conflict influences PTSD symptomology through exposure to bullying, which differs based on levels of neuroticism. The coefficients of each pathway are depicted in Figure 2 below. The interaction on the ‘a’ path was significant (*p* = 0.01), with a positive coefficient, demonstrating that when neuroticism levels are higher, the indirect effect of role conflict and exposure to bullying on PTSD symptomology is strengthened. A visual representation of the moderating effects can be seen in Figure 3 below. Furthermore, the R² change showed that 3% of the variance in the indirect effect is explained by the interaction of role conflict and neuroticism.

### 4.2. Moderated Mediation with Managerial Competencies as the Moderator

**Figure 4:**
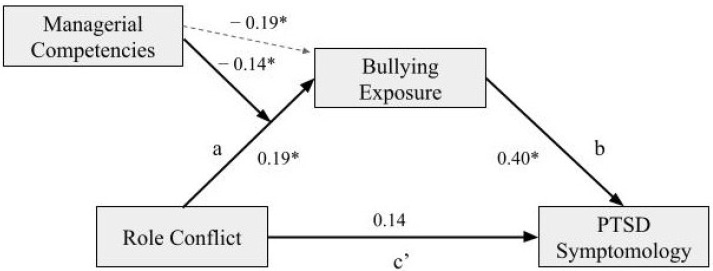
Coefficients of the moderated mediation model with neuroticism as a moderator. *Note.* * indicates *p* < 0.05. c’ (c-prime) refers to the direct effect.

**Figure 5:**
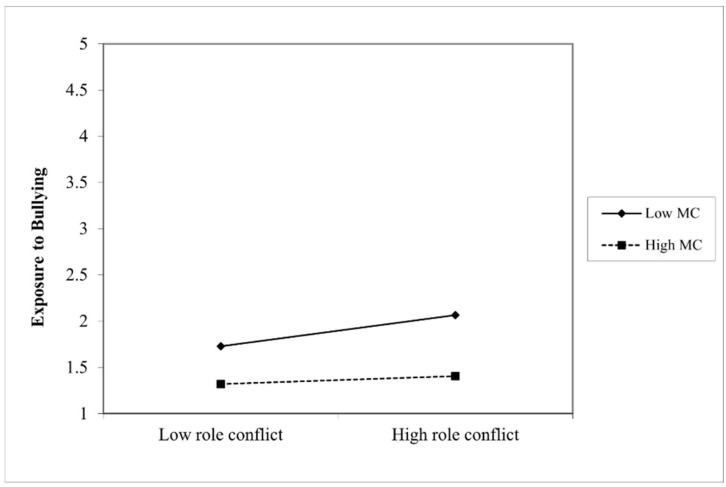
The moderating effects of managerial competencies on the indirect effect of role conflict and exposure to bullying. *Note:* MC stands for managerial competencies.

Additionally, the analysis found a statistically significant moderated mediation, with the moderator of managerial competencies reporting an index of −0.06 and bootstrapping confidence intervals between −0.13 and −0.01. Again, a full mediation was found where role conflict influences PTSD symptomology through exposure to bullying, which differs based on the level of managerial competencies. The coefficients of each pathway are depicted in Figure 4 below. The interaction on the ‘a’ path was significant (*p* = 0.00), with a negative coefficient, demonstrating that when managerial competencies are higher, the indirect effect of role conflict and exposure to bullying on PTSD symptomology is weakened. A visual representation of this moderating effect can be seen in Figure 5 below. Furthermore, the R² change showed that 5% of the variance in the indirect effect is explained by the interaction of role conflict and managerial competencies.

## 5. Discussion

The results of this study confirm that undergoing role conflict can lead to the experience of bullying in the workplace and, through that experience, the exhibition of PTSD symptomology. The results of full mediation demonstrate that role conflict in itself does not lead to PTSD symptomology but rather to workplace conflict, such as bullying, which, in turn, can lead to PTSD symptomology. Previous research, with few exceptions, has mainly investigated the relationship between role stressors such as role conflict and bullying (see [28]) and between bullying and PTSD (see [32]) separately, i.e., without examining the full chain of relationships that may link poor work environmental conditions (i.e., role conflict) with very serious health effects such as PTSD symptoms via the occurrence of bullying. Very recently, the need to dedicate more research to the study of processes and mechanisms linking bullying with its antecedents and consequences has been emphasized [47]. The present study contributes to filling this gap in the field, showing that role conflict may indeed fuel victimizing relationships at work that are conducive to debilitating mental health problems, bringing new original evidence on the traumatic potential of bullying and the process through which this occurs.

Furthermore, the results demonstrate that having higher levels of neuroticism strengthens the previously discussed relationship, ultimately resulting in those with neuroticism experiencing more PTSD symptomology under the same role conflict conditions as a result of the strengthened relationship of the indirect effect of role conflict and bullying. The graphical representation of this moderating effect also reveals that those with higher levels of neuroticism experience more exposure to bullying even at low levels of role conflict. Additionally, individuals with managers who possess higher levels of competencies experience a weakening of the aforementioned relationship, meaning that these individuals experience lower levels of PTSD symptomology due to the weakened relationship of the indirect effect. In fact, the graphical representation of this moderating effect revealed almost no difference in exposure to bullying even when role conflict was high for those who had managers with high competencies. In addition, those with managers with low competencies still experienced more bullying than those with managers with high competencies, even when role conflict was low. The full mediation found in both models shows that only through exposure to bullying does role conflict have an effect on PTSD symptomology, suggesting that other workplace stressors such as role ambiguity and work overload (discussed in the introduction) can also fit into this model as independent variables.

Indeed, similar studies investigating workplace bullying from the passive perspective have found other work environment stressors, such as job demands, that positively relate to bullying victimization [48]. Furthermore, studies exploring workplace bullying from the active perspective have also found that work environment factors such as organizational justice, organizational trust, and workaholism are also related to bullying perpetration [49,50]. As this study has found that managerial competencies, which alter and improve the interaction of employees with their work environment, moderate employee stress from poor working environments, managerial competencies may not just decrease the frequency of employees being bullied but may also decrease the frequency of which employees bully. In fact, managerial competencies have recently been found to positively relate to the psychological wellbeing of employees, suggesting that managerial competencies may act as the main factor in the occurrence (or nonoccurrence) of bullying, regardless of the work environment [38]. This finding is particularly relevant in light of studies that have found incidences of bullying even in optimal working environments such as HPWC, where bullying can still lead to organizational consequences such as decreased employee engagement [51]. The evidence of managerial competencies as the main factor in the bullying process, paired with the significant finding of managerial competencies in this study as a moderator in the investigated bullying process, provides strong evidence for the focus on managerial competencies as a basis for workplace bullying prevention and intervention.

## 6. Conclusions

### 6.1. Practical Implications

In the past, organizations have been aware that role conflict can lead to certain negative consequences, such as a decrease in job attitudes, namely, job satisfaction, organizational commitment, and employee engagement, which ultimately impact performance [52]. However, this present study reveals that role conflict can also put employees at risk of exposure to bullying, which, in turn, leads to mental health consequences, particularly PTSD symptomology. Furthermore, the present study has identified certain groups of individuals who could be at higher risk for these situations and a buffer to work-related stress, which can aid in the development of organizational intervention and prevention techniques.

Firstly, the present study has identified that individuals with neuroticism are not only more likely to experience workplace bullying in general but are even more so when role conflict in the workplace is high. Organizations should continue to focus on creating roles that are clear, where tasks do not contradict the position itself or place the employee in an unethical situation that could lead to cognitive dissonance. Decreasing role conflict would not only lead to better performance, as the literature has found in the past, but also to better employee wellbeing and positive work environments, as found in this study. Additionally, as personality tests are often part of recruitment and onboarding efforts, organizations should ensure that extra resources are available to hires in general, particularly for those who possess neurotic traits, to help reduce or control stress generated from role conflict or other sources of workplace stress that could lead to the experience of workplace bullying. These resources include but are not limited to stress reduction services such as mindfulness sessions, which have been associated with psychological needs satisfaction and stress reduction, specifically in those with neuroticism [53]. Other resources may include company structure organization, such as the formation of work teams or the assignment of mentors, which one study found to be a buffer to work stress for those with high neuroticism [54]. In addition, previous literature has found positive links between both peer and managerial support as well as training programs and their ability to diminish the effects of workplace stress, such as role conflict and/or bullying, independent of personality traits [55,56].

Another consideration for organizations revealed by the present study is the importance of the role of managers in moderating the workplace stress of their employees. This study has demonstrated that employees with competent managers not only experience less workplace bullying in general but also in the presence of other workplace stressors such as role conflict. Studies have found that training managers in active listening and positive feedback reduces employee stress [57]. Additionally, the decades-old Grid managerial training has been connected to positive employee benefits [58]. Managers who attended the Grid training have experienced positive organizational outcomes with their employees, such as improved performance, job satisfaction, and increased motivation and creativity [58]. These positive outcomes would not be possible if work stress were high. The results of a study analyzing rigorous managerial training programs across a two-year study found that management training programs lead to greater employee satisfaction and a reduction in employee turnover, justifying the cost of the training [59]. While not directly connected to stress reduction, reduced turnover and increased employee satisfaction could be related to less employee stress as a result of well-trained managers [59]. Other studies have found that the degree of perceived work stress is related to the type of leadership employed by managers and that transformational leadership has the best outcomes for employee stress [60,61]. The four “I′s” of transformational leadership sum up the key characteristics possessed by transformation leaders: idealized influence, inspirational motivation, intellectual stimulation, and individual consideration [60]. Interactive workshops for transformational leadership focusing on these four “I′s” have been successful in both the US and Canada and have been adapted in Europe [60].

The aforementioned managerial training programs are not the only programs connected with positive psychosocial health outcomes for employees. Indeed, the programs mentioned are older methods, which highlight the value, along with the findings of this study, of researching and developing more modern managerial competencies training programs that focus particularly on the management and reduction of employee stress. In any case, organizations should invest in training employees in supervisory positions and create robust recruitment standards for upper-level positions as competent managers lead to less-stressed employees and thus better organizational outcomes [4,5,13,61,62].

### 6.2. Limitations

When conducting studies on workplace bullying, it is important to take into consideration the possibility of bias in the reported data. As this study used the S-NAQ, which measures passive forms of bullying, that is, bullying as reported by the victims, several forms of bias may have occurred. Jenkins and colleagues [63] conducted a bullying allegations study, interviewing accused bullies. An analysis of the interview data found that in some cases, reasonable managerial action was interpreted by victims as bullying, as were certain justified organizational practices [63]. Like any subjective and self-reported variable, the results must be viewed with caution due to common method variance. However, it is difficult, if not impossible, to collect unbiased data when measuring workplace bullying as observation data may be equally affected by bias; that is, observers may only record bullying that is overt in nature, missing more subliminal forms that may make up the majority of workplace bullying in some instances [64]. Furthermore, bullying from the active perspective relies on participants truthfully reporting their mistreatment of others.

This study utilized a sample of workers from a large social cooperative organization. Thus, an additional limitation of this study is the lack of generalizability for the causes, effects, and moderators of workplace bullying in other sectors. Future research will have to confirm if the results found in this study are also true across other occupations, particularly in high-stress and high-demand sectors such as the medical field [3]. In addition, as mentioned previously, the significant findings of this study were based on a cross-sectional design. Therefore, the temporal link and causal conclusions between role conflict, exposure to bullying, and PTSD symptomology cannot be determined [65]. This is the most important limitation of this study, as a main element of bullying is the repeated pattern of mistreatment over time. However, longitudinal data can provide different limitations. For example, longitudinal studies are costly and pose difficulties when the correct lag times between variables are undetermined, such as in this study [65]. In this case, it then becomes complicated to assess if changes in one construct lead to changes in another if the appropriate time points for assessment are not precise [65]. In addition, you must control for external events occurring in the lag time that could have an effect on the measured variable [65]. For example, when assessing if role conflict measured at time A affects the experience of workplace bullying at time B in a particular company, if, between time A and time B, the participants’ company undergoes downsizing, without controlling for this factor, it would be impossible to know if stress from role conflict contributed to the experience of workplace bullying or if stress from the downsizing of the company did. Additionally, as this study explored for the first time the relationship between role conflict and PTSD symptomatology, it was important to have an idea of the relationship before conducting a more robust and costly study. Therefore, while subject to limitations, the cross-sectional design of this study was chosen with the purpose of the research and the constraints of the variables selected in mind.

Lastly, it is important to note that the assumption of normality was not adequately met. This was more or less expected as the questionnaires, such as the S-NAQ and the Civilian PTSD Checklist, rarely result in normally distributed answers due to the nature of their investigation. However, this unmet assumption does not affect the percent of variance explained; thus, the results are still significant contributions to research. Going forward, we will use more vigorous measures such as robust maximum likelihood in order to address the assumption of normality. Additionally, certain control variables should be considered in the future. Going forward, traumatic events in the last 12 months, gender, age, and personality should be controlled in order to ensure that the results are not due to confounding variables.

### 6.3. Future Implications

The significant moderating effects of managerial competencies on the indirect path between role conflict and bullying serve as empirical evidence for the social support theory. The social support theory tells us that social support contributes to health and wellbeing by protecting people from the adverse effects of stress. The theory claims that the surrounding social support can lead to wellbeing by acting as a moderator of stress and by promoting self-esteem and self-regulation [66]. Indeed, this study found this moderating stress effect to be true. However, what the present study did not investigate was the possible moderating effects of peer social support. Just as trained managerial competencies may be an organizational intervention for workplace bullying, perhaps employees can be trained in a similar manner. In fact, Ng and colleagues [67] recently developed a bystander behavior scale for the context of workplace bullying in the UK. The scale aims to assess the action or inaction of employees in the workplace who encounter the bullying of their colleagues. With this assessment, one can investigate whether bystanders′ responses influence outcomes for those exposed to bullying. Ng and colleagues [67] proposed their bystander behavior scale and study based on the Job-Demands Resource theory, proposing that passive and active bystanders can worsen or buffer, respectively, the effects of bullying on victim wellbeing. That is, passive bystanders can act as further demands for targets to cope with and active bystanders can act as a resource. To evaluate this proposal in the Italian context, this study could be repeated using bystander behavior as a moderator on the indirect path of workplace bullying and PTSD symptomology in order to investigate if bystander action weakens the relationship.

Lastly, although we have identified an important antecedent to workplace bullying as well as an important mental health consequence and two significant moderators, this study continues to view workplace bullying and PTSD symptomology in a between-subjects, cross-sectional manner. Therefore, there is still more to be investigated, such as how employees are individually affected by workplace bullying and how this could change over time. A further study exploring these phenomena, using longitudinal data to investigate the within-subjects results and the role of time, could serve as a future research consideration. Finding new methods to test the same or similar hypotheses that produce consistent results increases the confidence in the significance and generalizability of the findings, a systematic process that is underused yet valuable in research [65].

Going forward, organizations should be aware not only of the stress brought on by organizational factors such as role conflict but also of the work environment that can be negatively altered due to these variables. Furthermore, while role conflict alone causes stress in employees, the escalation to bullying has even more detrimental effects, such as PTSD symptomology. In order to protect the wellbeing of employees and, in turn, their performance, productivity, and creativity, organizations should focus on factors that can stop workplace bullying from happening in the first place, as prevention is much easier than intervention. This study has identified two principal factors for workplace bullying prevention: first, the importance of training managers in stress management competencies; second, providing adequate resources to employees to handle stress, particularly to those who are predisposed to maladaptive coping mechanisms (those with neuroticism). Additionally, role conflict should be avoided, as well as other workplace stressors; while not explicitly examined in this study, other workplace stressors such as role ambiguity and work overload can create the same escalated stress that can lead to bullying.

## Figures and Tables

**Table 1:** Means, standard deviations, and correlations with confidence intervals.

Variable	M	SD	1	2	3	4
1. Role conflict	2.46	0.89				
2. Bullying exposure	1.47	0.48	0.48 **			
			[0.38, 0.57]			
3. PTSD symptomology	2.06	0.91	0.24 **	0.28 **		
			[0.08, 0.38]	[0.12, 0.43]		
4. Neuroticism	2.45	0.86	0.19 *	0.23 **	0.32 **	
			[0.04, 0.34]	[0.10, 0.36]	[0.17, 0.45]	
5. Managerial competencies	3.35	0.82	−0.31 **	−0.43 **	−0.15	−0.40 **
			[−0.46, −0.15]	[−0.55, 0.30]	[− 0.31, 0.04]	[−0.52, −0.28]

*Note.* Values in square brackets indicate the 95% confidence interval for each correlation. * indicates *p* < 0.05. ** indicates *p* < 0.01.

## Data Availability

The SPSS file of raw data can be obtained upon reasonable request from the first author.

## Appendix A

**Table A1:** Factor loadings for the total measurement model.

Items	Factor Loadings
Role Conflict	
1. I work with two or more groups who operate quite differently	0.32
2. I have to buck a rule or policy to carry out an assignment	0.54
3. I receive incompatible requests from two or more people	0.80
4. I receive an assignment without adequate resources and materials to execute it	0.76
5. I work on unnecessary things	0.75
Neuroticism	
6. I tend to get stressed out easily	0.80
7. I often feel down	0.76
8. I tend to get worried easily	0.75
9. I am hurt easily	0.64
PTSD Symptomology	
10. Memories, thoughts or images	0.81
11. Upset when reminded	0.93
12. Avoid activities or situations	0.52
13. “Superalert”	0.62
Exposure to Bullying	
14. Someone withholding information which affects your performance	0.65
15. Spreading of gossip and rumors about you	0.50
16. Being ignored, excluded or being ‘sent to Coventry’	0.74
17. Having insulting or offensive remarks made about your person (i.e., habits and background), your attitudes or your private life	0.79
18. Being shouted at or being the target of spontaneous anger (or rage)	0.59
19. Repeated reminders of your errors or mistakes	0.76
20. Being ignored or facing a hostile reaction when you approach	0.72
21. Persistent criticism of your work and effort	0.69
22. Practical jokes carried out by people you don′t get on with	0.59
Managerial Competencies	
23. When necessary, my supervisor will stop additional work being passed on to me	0.45
24. My supervisor reviews processes to see if work can be improved	0.85
25. My supervisor prioritizes future workloads	0.70
26. My sup. deals objectively with employee conflicts	0.80
27. My sup. deals with employee conflicts head on	0.78
28. My sup. acts as a mediator in conflict situations	0.78
29. Supports employees through incidents of abuse	0.77
30. My sup. follows up conflicts after resolution	0.81
31. My sup. makes it clear he or she will take ultimate responsibility if things go wrong	0.76
